# Influence of the Mixture of Carrageenan Oligosaccharides and Egg White Protein on the Gelation Properties of *Culter alburnus* Myofibrillar Protein under Repeated Freezing–Thawing Cycles

**DOI:** 10.3390/antiox11010032

**Published:** 2021-12-24

**Authors:** Zhongli Zhang, Zhouyi Xiong, Noman Walayat, Jose M. Lorenzo, Jianhua Liu, Asad Nawaz, Hanguo Xiong

**Affiliations:** 1 College of Food Science and Technology, Huazhong Agricultural University, Wuhan 430070, China; zhangzhongli@webmail.hzau.edu.cn; 2 Fisheries Research Institute, Wuhan Academy of Agricultural Sciences, Wuhan 430207, China; 3 College of Food Science and Technology, Zhejiang University of Technology, Hangzhou 310014, China; noman.rai66@gmail.com (N.W.); jhliu@zjut.edu.cn (J.L.); 4 Centro Tecnológico de la Carne de Galicia, Avd. Galicia nº 4, Parque Tecnológico de Galicia, San Cibrao das Viñas, 32900 Ourense, Spain; jmlorenzo@ceteca.net; 5 Área de Tecnología de los Alimentos, Facultad de Ciencias de Ourense, Universidad de Vigo, 32004 Ourense, Spain; 6 Jiangsu Key Laboratory of Crop Genetics and Physiology, College of Agriculture, Yangzhou University, Yangzhou 225009, China; 007298@yzu.edu.cn

**Keywords:** carrageenan oligosaccharide, egg white protein, gelation properties, myofibrillar protein, *Culter alburnus*

## Abstract

This study aims to investigate the influence of the mixture (CGO/EWP) of carrageenan oligosaccharide (CGO) and egg white protein (EWP) (CGO/EWP, CGO: EWP = 1:1, m/m) on the functional, structural, and gelling properties of *Culter alburnus* myofibrillar protein (MP) during repeated freezing–thawing cycles by treating MP samples separately with EWP, CGO, or CGO/EWP based on the wet weight (1%, m/m), using samples without any cryoprotectant as the blank group. After the second repeated freezing–thawing cycle, the sulfhydryl group content was found to be significantly (*p* < 0.05) higher in the CGO/EWP (30.57 nmol/mg) and CGO (36.14 nmol/mg) groups than in the EWP group (23.80 nmol/mg), indicating that CGO/EWP and CGO can more effectively delay the oxidative deterioration of functional groups. Additionally, the surface hydrophobicity was shown to be significantly lower in the CGO (25.74) and CGO/EWP (27.46) groups than in the EWP (34.66) and blank (39.32) groups. Moreover, the α-helix content was higher in the CGO (35.2%) and CGO/EWP (32.3%) groups than in the EWP (29.2%) and blank (25.0%) groups. These data indicated that CGO and CGO/EWP could more effectively increase the structural stability, thereby inhibiting the exposure of hydrophobic groups and curbing the decline of α-helix content. During the heat-induced gel-forming process, EWP and CGO/EWP could enhance the gel viscoelasticity and strength. After the second freezing–thawing cycle, when compared with the blank group, the CGO/EWP group showed significantly (*p* < 0.05) higher water-holding capacity (66.30% versus 53.93%) and shorter T_22_ relaxation time (413.56 versus 474.99 ms). The integrated results indicated that CGO/EWP could more effectively delay the decrease of protein–water molecular interaction forces in the MP gel. This study shed light on the mechanism of CGO/EWP as a cryoprotective mixture in improving the deterioration of MP gelation properties during repeated freezing–thawing cycles.

## 1. Introduction

As a popular freshwater fish species in China, *Culter alburnus* exhibits remarkable nutritional value for commercial processing. Recently, there has been a rapid increase in the tonnage of frozen-processed *Culter alburnus* for transportation and long-term preservation. Unfortunately, temperature fluctuations can occur in the incomplete cold chain in commercial transportation, accelerating the oxidation of proteins, increasing the volume of ice crystals, and deteriorating the quality of fish muscle products, such as reduced texture and juiciness [1]. Moreover, repeated freezing and thawing is usually inevitable in the retail sector [2].

The functional groups and structural properties of myofibrillar protein (MP), a main protein in muscle proteins (55–65%), play a vital role in the quality of fish muscle products [3,4,5]. However, long-term freezing or repeated freezing–thawing cycles (F-T-Cs) cause the oxidation deterioration of MP functional groups and structural changes, such as the oxidation of sulfhydryl groups and the increased hydrophobicity of protein molecules, disrupting the ideal protein–protein and protein–water intermolecular interactions in heat-induced MP gels, loosening the three-dimensional gel network, reducing the gel viscoelasticity and strength, and negatively affecting gel properties [6]. Therefore, in the food industries, cryoprotectants (such as sucrose and sorbitol) are usually added in the fish muscle protein products (such as surimi, meat balls, etc.) to reduce the oxidation degradation of functional groups and enhance the MP structural stability during long-term freezing or repeated F-T-Cs [2,7]. The cryoprotectant mechanism can be divided into two types: (i) inhibiting the formation of ice crystals around the protein surface of muscles or enhancing the antioxidant capacities by its specific chemical groups, such as some organic acids, oligosaccharides and polyphenols) and (ii) promoting the formation of compact gel network through their filling effects, such as egg white protein, starch, etc. [8,9]. At present, most studies focus on the potential of a new single substance as a cryoprotectant and ignore the possibility of combining the mechanism of two different substances as a cryoprotectant.

As a common additive in aquatic products, egg white protein (EWP) has the advantages of a wide source range and low price, and it is usually used as an aquatic product emulsifier, foaming agent, and gel-softening enzyme inhibitor [10,11]. Importantly, EWP can be used as a filler to promote the gel-forming ability of fish muscle products, but its effect is still limited in suppressing MP oxidation deterioration during long-term frozen storage or repeated F-T-Cs [10,12,13].

Carrageenan oligosaccharides (CGO), composed of carrageenan extracted from various red seaweed species, have a vital capability in inflammation, cell growth, and immune defense [14]. Furthermore, CGO shows effective antioxidant activity as a cryoprotectant to protect MP during long-term frozen storage or repeated F-T-Cs [2,15]. It is well-known that EWP and CGO have different mechanisms for improving the MP gel quality during long-term freezing or repeated F-T-Cs, but to our knowledge, no study has ever explored the synergistic effect of the different mechanisms of CGO and EWP and compared their combined effect with the effect of adding EWP or CGO alone.

Therefore, this study aimed to enrich the understanding of the mechanisms of CGO and EWP mixture (CGO/EWP) in protecting the MP gelation performance by comparing the effects of adding CGO/EWP, CGO, or EWP alone on the functional, structural, and gelling properties of different treated MP samples during repeated F-T-Cs). The results provide useful information for the potential use of CGO/EWP in preventing the oxidative, nutritional, and sensory deterioration of fish muscle protein products (such as fish ball, fish cakes, and crab sticks made of surimi) under repeated F-T-Cs or long-term frozen storage.

## 2. Materials and Method

### 2.1. Materials

The Wuhan Academy of Agricultural Sciences provided *Culter alburnus* (weight: 2.5 ± 0.52; n = 18) by storing the live fishes in crushed ice and transporting them immediately to our lab. Back muscles were minced to surimi for MP preparation. Eggs were purchased from the local market of Huazhong Agricultural University (Wuhan, China). Carrageenan oligosaccharide ([C6H9O8Na]n, n =2–10,500–5000 Da) was provided by BZ. Oligo biotech Co., Ltd., (Qingdao, China).

### 2.2. Methods

#### 2.2.1. Preparation for the Mixture of Carrageenan Oligosaccharide and Egg White Protein (CGO/EWP)

Briefly, the egg white was separated from the egg, followed by freeze-drying to obtain the powder as egg white protein (EWP). Next, the EWP and CGO (mass ratio = 1:1, *w*/*w*) were mixed in distilled water at the final concentration of 10% (*W*/*W*) and pH was adjusted to 7.0 with 1 mol/L hydrochloric acid or sodium hydroxide. Finally, the mixed solution was stirred with a magnetic treatment mixer (2 h, 4 °C) and freeze-dried to obtain the CGO/EWP mixture.

#### 2.2.2. Extraction of Myofibrillar Protein and Preparation of Samples

The extraction of MP from surimi followed our previous method [3]. Briefly, low salt buffer (0.05 mol/L of NaCl, 20 mmol/L of PBS, pH = 7.5) and high salt buffer (0.6 mol/L of NaCl, 20 mmol/L of PBS, pH = 7.5) were prepared and stored for 12 h at 4 °C, followed by mixing 100 g of surimi with 500 mL of cooled low salt buffer using a homogenizer (4 °C, 1 min, 5000 rpm) (XHF-DY, Ningbo Scientz Biotechnology Co., Ltd., Ningbo, China) and centrifugation to collect the precipitation. Then, the precipitate was homogenized (4 °C, 15 min, 8000× *g*) with 5 volumes of low salt solution (m/v) and centrifuged again to collect the precipitation. This procedure was repeated twice. Next, 100 g of the obtained precipitate was homogenized (4 °C, 1 min, 5000 rpm) with 500 mL of cooled high salt buffer and placed at 4 °C for 12 h to completely dissolve the MP, followed by centrifugation (4 °C, 15 min, 8000× *g* ) to retain the supernatant, mixing the supernatant with 10 volumes of cooled deionized water and centrifugation to obtain the precipitate, which was defined as the MP sample. Finally, the protein concentration in the precipitate was determined by the Biuret method [16] and used as the MP concentration in MP samples.

The addition amount of EWP, CGO, and CGO/EWP was controlled as reported by Nikoo, et al. [17]. Based on their wet weight, MP samples were supplemented separately and mixed uniformly with 1% EWP, CGO, or CGO/EWP (*w*/*w*). The samples without EWP, CGO, or CGO/EWP were defined as the blank group.

All the samples were frozen at −18 °C for 42 h and thawed in 20 °C for 6 h, which was defined as one F-T-C. In this study, MP samples were analyzed at 0, 1, 2, 3, and 4 F-T-Cs.

#### 2.2.3. Sulfhydryl Group Content

Analysis of the sulfhydryl group content followed the Ellman’s method [18] with minor modifications. Briefly, 0.6 mol/L of NaCl was used to dilute the MP sample to 5 mg/mL, followed by mixing the MP (1 mL) with 9 mL of buffer (containing 0.6 mol/L of NaCl, 8 mol/L of Urea, 2% SDS, 10 mmol/L of EDTA, pH = 7.0), and adding 1 mL of 0.1% DTNB reagent (5,5′-dithiobis (2-nitrobenzoic acid)) (containing 0.2 mol/L of Tris-HC1, pH = 8.0). Finally, the mixed solution was reacted at 40 °C for 30 min and the absorbance at 412 nm was measured using an Ultraviolet spectrophotometer (TU-18, Beijing Purkinje General Instrument Co., Ltd., Beijing, China), with each sample measured three times in triplicate. Molar extinction coefficient 136,000 M^−1^ cm^−1^·cm/L was used to calculate the sulfhydryl group content by Equation (1).

(1) $$\mathrm{Sulfhydryl}\;\mathrm{group}\;\mathrm{content}\left(\mathrm{nmol}/\mathrm{mg}\right)=\frac{{\mathrm{Absorbance}}_{\left(412nm\right)}*A}{B*C}$$

where A indicates dilution factor; *B*, molar extinction; *C*, MP concentration (mg/mL).

#### 2.2.4. Carbonyl Group Content

The carbonyl group content was determined by 2,4-dinitrophenylhydrazine (DNPH) as previously reported [19]. For protein precipitation, MP was diluted to 5 mg/mL with 0.6 mol/L of NaCl and added 10% trichloroacetic acid (TCA), followed by centrifugation (4 °C, 5000× *g*, 5 min) to obtain the precipitate, adding 8 mL of DNPH (2 g/L, containing 2 mol/L of HCl, reaction at 25 °C for 60 min in the dark, and shaking the sample every 10 min. Next, the protein was precipitated in the mixed solution with 20% Trichloroacetic acid, followed by centrifugation (4 °C, 5000× *g*, 5 min), washing the precipitate three times with 1 mL ethyl acetate: ethanol mixture buffer (1:1, *v*/*v*) (to remove excessive DPNH) until the solution was colorless. Subsequently, the precipitate was re-dissolved in 2 mL of 6 mol/L of guanidine hydrochloride (containing 20 mmol/L of PBS, pH = 6.5) and reacted at 4 °C in the dark for 12 h. Finally, the absorbance of each treated sample at 412 nm was measured using a UV spectrophotometer, with three measurements for each sample in triplicate. The 22,000 (mol/L)^−1^ cm^−1^ was used as the protein absorption amount to calculate the carbonyl content by Equation (2):

(2) $$\mathrm{Carbonyl}\;\mathrm{group}\;\mathrm{content}\left(\mathrm{nmol}/\mathrm{mg}\right)=\frac{{\mathrm{Absorbance}}_{\left(412\mathrm{nm}\right)}*A}{B*C}$$

where A indicates the dilution factor; B, protein absorption amount; C, MP concentration (mg/mL).

#### 2.2.5. Dityrosine Content

The dityrosine content in the MP sample was determined using a previous method [15] with a slight modification. Briefly, 0.6 mol/L NaCl was used to dilute the MP sample to 1 mg/mL and the dityrosine fluorescence intensity in the solution was evaluated by a fluorescence spectrophotometer (F-4600, Hitachi High Technologies Corporation, Tokyo, Japan) at the excitation and emission wavelength of 325 and 420 nm, and the slit width of 10 nm, with three measurements for each sample in triplicate. The fluorescence intensity result was defined as the MP dityrosine content in the unit of A.U.

#### 2.2.6. Surface Hydrophobicity (S_0_)

The surface hydrophobicity (S_0_) of MP samples was measured as reported by Lin et al. [20] with slight modifications. Briefly, 0.6 mol/L of NaCl was used to dilute MP to different concentrations (0.2, 0.3, 0.5, and 1 mg/mL), followed by mixing 40 μL of ANS solution (2 mmol/L, containing 0.2 mol/L of PBS, pH = 7.5) with 10 mL of diluted MP sample and reaction at 25 °C for 30 min in the dark. Finally, a fluorescence spectrophotometer was used to determine the mixture fluorescence intensity at the emission and excitation wavelength of 470 and 390 nm, and the slit width of 5 nm, with three measurements for each sample in triplicate. The surface hydrophobicity (S_0_) result was calculated by the linear slope between fluorescence intensity and corresponding MP concentrations by Equation (3):

(3) $$\mathrm{Surface}\;\mathrm{hydrophobicity}(S_{0})=\frac{n\Sigma \left(X*Y\right)-\left(\Sigma X*\Sigma Y\right)}{{n\Sigma X}^{2}-{\left(\Sigma X\right)}^{2}}$$

where X indicates the MP concentration (mg/mL); Y, the fluorescence intensity corresponding to MP concentration; n: quantity of diluted gradient concentrations (0, 0.2, 0.3, 0.5, and 1 mg/mL) (n = 5).

#### 2.2.7. Endogenous Fluorescence Intensity

The measurement of endogenous fluorescence intensity (EFI) followed the method of Zhang, Xiong, Lu, Walayat, Hu, and Xiong [3]. Briefly, 0.6 mol/L NaCl was used to accurately adjust the MP sample concentration to 1 mg/mL and the EFI values were estimated using a Fluorescence spectrophotometer at the emission wavelength of 300–400 nm, the excitation wavelength of 295 nm, and the scanning speed of 1200 nm/min. The highest peak value obtained at around 335 nm was used as the EFI of the MP sample.

#### 2.2.8. Circular Dichroism

The MP secondary structure was measured by circular dichroism (J-1500-150, JASCO corporation 192–8537, Tokyo, Japan) as reported by Xiong et al. [21]. Briefly, 0.6 mol/L NaCl was used to dilute the MP samples to 0.5 mg/mL. Quartz cell (0.1 cm) added with 0.6 mol/L of NaCl was used as the blank baseline. Circular dichroism was performed in the scanning wavelength range of 200 to 250 nm, the width of 1 nm, and the scanning speed of 200 nm/min, and each sample was scanned three times. The protein concentration was calculated using 110 g/mol, and the MP secondary structure was estimated using the Young’s model in the software of the circular dichroism analyzer.

#### 2.2.9. Preparation of Heat-Induced MP Gel

Considering that EWP can promote gel formation as a filler in the MP gel network, MP gel properties (such as dynamic rheology, gel strength, LF-NMR) were determinized without removing EWP.

The heat-induced MP gel was prepared as reported by Zhang, Xiong, Lu, Walayat, Hu, and Xiong [3]. After adjusting the sample NaCl concentration to 0.6 mol/L and MP concentration to 60 mg/mL by NaCl reagent and PBS buffer (20 mmol/L, pH = 7.5), 60 mg/mL of MP sample was prepared in a cylindrical container (diameter 32 mm, height 22 mm) or in a glass tube (diameter 11 mm, height 80 mm).

The preparation procedure of heat-induced gel consisted of equilibration at 40 °C for 30 min, followed by heating at 80 °C for 30 min, then immediate cooling in crushed ice for 10 min, and finally storage at 4 °C for 12 h.

#### 2.2.10. Dynamic Rheological Analysis

According to our previous method [3], a rheometer (DHR2 rotational rheometer, TA instruments Crawley, United Kingdom) was used to evaluate the temperature sweep mode changes of MP samples in dynamic rheology. Briefly, 0.6 mol/L of NaCl was used to adjust the MP concentration to 60 mg/mL, followed by placing ~1 mL MP sample on the plate equipped with a temperature controller, adjusting the gap to 1 mm from the geometry upper plate (aluminum parallel-plate with a 40-mm diameter), and coating the edges with glycerol to prevent the sample from evaporation. The test was performed in the temperature range of 25 to 90 °C at a heating rate of 2 °C/min and the strain and frequency of 0.1% and 0.1 Hz. Finally, the rheological results were expressed as storage modulus (G’) and loss modulus (G”).

#### 2.2.11. Gel Strength

The MP gel strength was analyzed using TA XT. plus texture analyzer (Stable Micro System, London, UK) according to Liu, et al. [22] at the probe (spherical, P/0.25 s) penetration depth of 10 mm (rate 1 mm/s), the trigger force of 5 g, and the return distance of 10 mm (rate 1 mm/s). Each sample was analyzed 3 times in triplicate. The gel strength was calculated by Equation (4):

(4) $$\mathrm{Gel}\;\mathrm{strength}\left(g*\mathrm{cm}\right)=\mathrm{Breaking}\;\mathrm{force}\left(g\right)*\mathrm{Breaking}\;\mathrm{distance}\left(\mathrm{cm}\right)$$

#### 2.2.12. Water-Holding Capacity

Changes in the water-holding capacity (WHC) of the heat-induced MP gel were investigated using a previously reported method [23]. First, the centrifuge tube weight (*W_1_*) was determined, followed by adding about 1 g of MP gel sample in the tube as weight (*W_2_*) and centrifugation to remove the water from the gel sample. Finally, the centrifuge tube and gel sample were weighed again as *W_3_*. In this test, three measurements were performed for each sample in triplicate, and WHC was calculated by Equation (5):

(5) $$\mathrm{WHC}\left(\%\right)=\frac{W_{2}-W_{1}}{W_{3}-W_{1}}$$

#### 2.2.13. T_2_ Relaxation Time

The T_2_ relaxation time of MP gel samples was determined as previously reported [3] with minor modifications. Briefly, the gel was formed in a glass tube and inserted into the low field-nuclear magnetic resonance (LF-NMR) analyzer (Niumag Electric Company, Shanghai, China) to test the T_2_ relaxation time at the resonance frequency of 125 K Hz, with magnetic field strength = 0.5 T, TW = 4000 ms, TE =0.4 ms, PRG = 2, NS = 8, and NECH = 15,000.

#### 2.2.14. Proton Density Image

Proton density imaging, a magnetic resonance imaging (MRI) technology, can reflect water distribution without damaging the sample [3]. LF-NMR was used to analyze the gel samples formed in the glass tube by scanning each sample at three layers with a 5-mm width and a 0.5-mm gap. Finally, the water distribution state of the gel samples was shown by the color scale in the proton density map for pseudo color processing of the gel images.

#### 2.2.15. MP Gel Selective Solubility

The gel selective solubility was measured as reported by PerezMateos, et al. [24] with some modifications. First, MP gel (1 g) samples were accurately weighed and homogenized (4 °C, 5000 rpm, 1 min) to full dissolution in 9 mL of different buffer solutions as shown below: 

Solution (A) = 0.05 mol/L NaCl

Solution (B) = 0.6 mol/L NaCl

Solution (C) = 0.6 mol/L NaCl + 1.5 mol/L Urea

Solution (D) = 0.6 mol/L NaCl + 8 mol/L Urea

After standing at 4 °C for 60 min, each sample was centrifuged (4 °C, 10,000× *g*, 15 min). Finally, the Lowry method [25] was used to measure the supernatant protein content. The distinction in the buffer soluble protein content was calculated as the non-covalent intermolecular interaction force and expressed in the unit of soluble protein mg/mL. The non-covalent intermolecular interaction contents were calculated separately by Equations (6)–(8):

Ionic bond content = Solution (B)–Solution (A)(6)

Hydrogen bond content = Solution (C)–Solution (B)(7)

Hydrophobic interaction content = Solution (D)–Solution (C)(8)

Each sample was measured in triplicate.

#### 2.2.16. Statistical Analysis

The data were processed by one-way ANOVA and Duncan’s multiple range test (with *p* < 0.05 as a significant difference) in the SPSS software 21.0 (IBM Corp, Armonk, New York, NY, USA). All the figures were generated using the origin software (8.5 Origin Lab Inc., Northampton, MA, USA) and all results are presented as mean ± standard deviation (SD) (n = 3 or 9).

## 3. Results and Discussion

### 3.1. Functional Group

**Figure 1:**
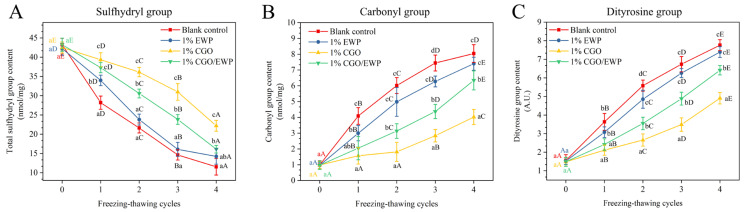
Changes of sulfhydryl group (**A**), carbonyl group (**B**), and dityrosine group (**C**) in *Culter alburnus* myofibrillar protein samples added with EWP, CGO, or CGO/EWP during repeated freezing–thawing cycles. The blank group was not added with EWP, CGO, or CGO/EWP. A significant difference (*p* < 0.05) between different experimental groups in the same freezing–thawing cycle is expressed by lowercase letters (a–c), and a significant difference (*p* < 0.05) between different freezing–thawing cycles in the same experimental group is expressed by different uppercase letters (A–E).

The MP gelation properties are closely related to the changes of the functional groups induced by various conditions [20], such as oxidation and icing, leading to the oxidation of sulfhydryl groups into inter/intra-molecular disulfide bonds, the formation of carbonyl groups between amino acid side chains, and the conversion of tyrosine to dityrosine [3,15]. Thus, the contents of carbonyl, sulfhydryl, and dityrosine are usually used as the indicators to reflect the degree of protein deterioration. As shown in Figure 1A, after the second F-T-C, the sulfhydryl content was significantly (*p* < 0.05) higher in the CGO group (36.14 nmol/mg) than in the CGO/EWP group (30.57 nmol/mg); however, their sulfhydryl content was both significantly (*p* < 0.05) higher than that of the EWP (23.80 nmol/mg) or blank (21.62 nmol/mg) groups. Meanwhile, as shown in Figure 1B,C, the content of carbonyl and dityrosine was higher in the CGO group (3.14 nmol/mg and 2.66 A.U.) than in the CGO/EWP group (1.81 nmol/mg and 3.55 A.U.), but both of their values were significantly (*p* < 0.05) lower than the values of the EWP (4.98 nmol/mg and 4.85 A.U.) and blank (6.01 nmol/mg and 5.58 AU) groups.

The formation of carbonyl groups and the conversion of sulfhydryl groups to disulfide bonds are related to protein oxidation, leading to MP fragmentation and irregular aggregation and thus the loss of the muscle product quality, such as texture deterioration, unpleasant flavor, and decreased nutritional value [21]. Compared with EWP, CGO showed a better protective effect on MP functional groups during repeated F-T-Cs. As previously reported, CGO can interact with myosin-charged amino acid polar residues to form hydrogen bonds and replace the myosin surface water molecules, thus reducing the volatility and flexibility of protein chains during freezing [26]. Meanwhile, CGO has the ability to scavenge free radicals and reduce the oxidation of polar amino acid residues on the peptide chains [27]. Therefore, CGO in the CGO/EWP mixture can be assumed to play a vital role in its higher efficiency than EWP in inhibiting the deterioration of MP functional properties. Furthermore, CGO also delayed the oxidation of sulfhydryl groups and inhibited the increase of carbonyl groups, contributing to reducing the weakening of MP’s ability to form a compact gel network [3,15].

### 3.2. Surface Hydrophobicity (S_0_)

**Figure 2:**
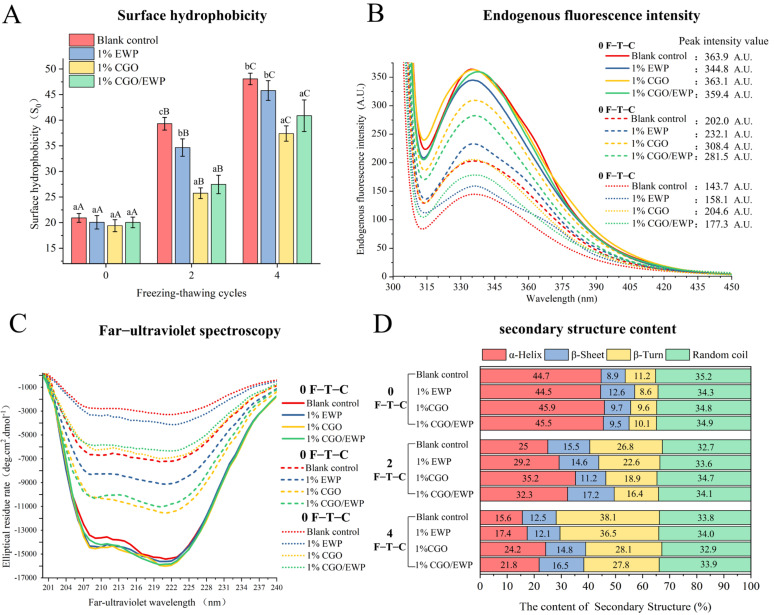
Changes of surface hydrophobicity (**A**), endogenous fluorescence intensity (**B**), far-ultraviolet spectroscopy (**C**), and secondary structure content (**D**) of *Culter alburnus* myofibrillar protein added with EWP, CGO, or CGO/EWP during freezing–thawing cycles. The blank group was not added with EWP, CGO, or CGO/EWP. A significant difference (*p* < 0.05) between different experimental groups in the same freezing–thawing cycle is expressed by different lowercase letters (a–c), and a significant difference (*p* < 0.05) between the different freezing–thawing cycles in the same experimental group is expressed by different uppercase letters (A–C).

Surface hydrophobicity can reflect the exposure degree of MP hydrophobic amino acids [3]. Naturally, hydrophobic amino acids exist in the protein folding core, but structural changes can expose hydrophobic amino acids to MP surfaces [3]. As shown in Figure 2A, with the increase of F-T-Cs, the surface hydrophobicity increased in the four groups, proving that repeated F-T-Cs led to the exposure of hydrophobic groups. After the second F-T-C, the surface hydrophobicity was significantly (*p* < 0.05) lower in the CGO (25.74) and CGO/EWP (27.46) groups than in the EWP (34.66) and the blank (39.32) groups.

Consistent with a previous report, CGO could interact with MP through hydrogen bonds to inhibit the exposure of MP polar amino acid residues [27]. Therefore, compared with EWP, the CGO in the CGO/EWP mixture could enrich its hydroxyl content, which can interact with MP to increase its structural stability. Meanwhile, the increase of surface hydrophobicity can enhance the irregular aggregation of MP and increase the hydrophobic interaction force between protein–protein molecules in the heat-induced MP gel, loosening the three-dimensional gel network and reducing the gel strength [3,20,28].

### 3.3. Endogenous Fluorescence Intensity

The inherent fluorescent properties of proteins are usually used to detect changes in the tertiary structure of proteins and evaluate the exposure of tryptophan residues to the hydrophilic microenvironment [29]. In Figure 2B, the endogenous fluorescence intensity (EFI) was seen to decrease in the four experimental groups with the increase of F-T-Cs. The surface hydrophobicity (Figure 2A) and EFI results proved that repeated F-T-Cs could cause the unfolding of the MP tertiary structure and the exposure of hydrophobic groups on the MP surface. Furthermore, repeated F-T-Cs could also cause MP unfolding and accelerate the oxidation of sulfhydryl groups to disulfide bonds and the formation of carbonyl groups between the amino acid side chains (Figure 1), resulting in irregular MP aggregation [29]. After the second F-T-C, the EFI value was higher in the CGO group than in the CGO/EWP group, but both of their EFI values were higher than the values of the EWP or blank group. The EFI results again demonstrated that CGO was more effective than EWP in enhancing the MP structural stability and delaying the deterioration of its functional properties during repeated F-T-Cs.

As previously reported [30], the hydrogen bond formed between the hydroxyl groups of oligosaccharides and proteins is one of the mechanisms by which oligosaccharides provide low-temperature cryoprotection. Similarly, Nikoo, Benjakul, and Rahmanifarah [17] reported that some substances with rich hydroxyl content (such as polyols, etc.) can interact with the hydrophobic groups inside the protein to increase the structural stability of the protein. Therefore, combined with the current results of surface hydrophobicity (Figure 2A) and EFI (Figure 2B), CGO has more hydroxyl groups than EWP, which can increase the phase incorporation with MP in F-T-Cs, thus reducing the exposure of MP hydrophobic residues and protecting its integrity in repeated F-T-Cs. Furthermore, based on data of sulfhydryl, carbonyl, and dityrosine contents (Figure 1), CGO in the CGO/EWP mixture plays a major role in delaying the oxidative degradation and enhancing the structural stability of MP in F-T-Cs, thus conducive to reducing the exposure of hydrophobic groups and inhibiting the decline of MP gelation properties [3].

### 3.4. Circular Dichroism

Circular dichroism is a useful tool for analyzing the MP secondary structural changes, which are also consistent with the changes of its functional properties [31]. In Figure 2C, two negative peaks at around 208 and 222 nm could be obviously observed in the far-ultraviolet spectra, which can reflect the MP α-helix content related to the n-π transition in the peptide bond backbone. At the 0 F-T-C, no significant difference was noted between the four groups in the far-ultraviolet spectra and the α-helix content. However, after repeated F-T-Cs, the four groups showed a decrease in the far-UV negative peak intensity, and a varying degree of reduction in the α-helical content. According to a previous report [3], MP oxidation can result the transformation of ordered α-helix to loose β-sheet and reduce the MP gelation properties. In Figure 2D, it was shown that after the second F-T-C, the α-helix content was higher in the CGO (35.2%) and CGO/EWP (32.3%) groups than in the EWP (29.2%) and blank (25.0%) groups, because CGO has a remarkable antioxidant activity against oxidative changes [15]. Besides, CGO can also improve the MP non-covalent bond stability and restrain the alterations of the secondary structure by interacting with MP and decreasing the reaction in the protein chain during F-T-Cs [26]. Therefore, based on the changes in the MP functional properties and structures, the CGO in the CGO/EWP mixture showed a higher efficiency than adding EWP alone.

### 3.5. Rheological Properties

**Figure 3:**
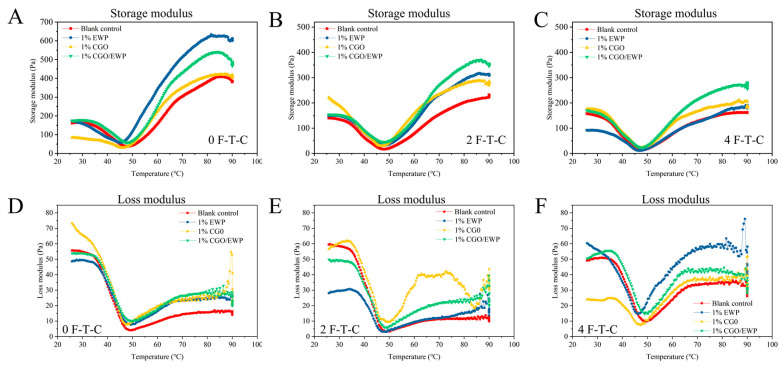
Changes of storage modulus (G′) (**A**–**C**) and loss modulus (G″) (**D**–**F**) of the temperature sweep of *Culter alburnus* myofibrillar protein added with EWP, CGO, or CGO/EWP during freezing–thawing cycles. The blank group was not added with EWP, CGO, or CGO/EWP.

The temperature sweep mode in the rheological properties can be used to evaluate the changes in the MP gel viscoelasticity through the variations of the storage modulus (G′) and loss modulus (G′′), which are associated with the network structure and intermolecular interaction in the heat-induced MP gel [3,32]. In Figure 3, the four experimental groups were seen to have a similar curve in G′ and G′′, with the G′ value always higher than the G′′ value, indicating that the typical viscoelastic behavior of MP gelation properties was maintained during repeated F-T-Cs [3]. At the 0 F-T-C, the values of G′ and G′′ were noted to be higher in the EWP and CGO/EWP groups than in the CGO and blank groups, which was related to the expansion of the globular protein in EWP (such as Ovalbumin) into a molten globular structure as a filler in the MP gel network during heating, leading to the increase of gel viscoelasticity [10,33,34].

After the fourth F-T-C, the four experimental groups showed a varying degree of decrease in the values of G′ and G′′, proving repeated F-T-Cs led to the deterioration of MP gelation properties and the decrease of gel viscoelasticity. However, in the second F-T-C, the values of G′ and G′′ were higher in the other three groups than in the blank group, indicating that adding CGO, EWP or CGO/EWP can reduce the decline of MP gel viscoelasticity. Besides, the values of G′ and G′′ were higher in the CGO group than in the blank group, indicating that CGO could protect the MP gelation properties during F-T-Cs by suppressing the oxidative deterioration of its functional groups (Figure 1) and increasing its structural stability (Figure 2). Furthermore, the values of G’ and G’’ were higher in the CGO/EWP group than in the CGO or EWP group, implying that the major role of CGO in the CGO/EWP mixture was to reduce the oxidative deterioration of MP functional groups and increase its structural stability, thereby suppressing the degradation of its gelation properties. Meanwhile, the vital role of EWP in the CGO/EWP mixture could be assumed to serve as a filler to improve the gelation properties already damaged by repeated F-T-Cs.

### 3.6. Gel Strength and Water Holding Capacity in MP Gel

**Figure 4:**
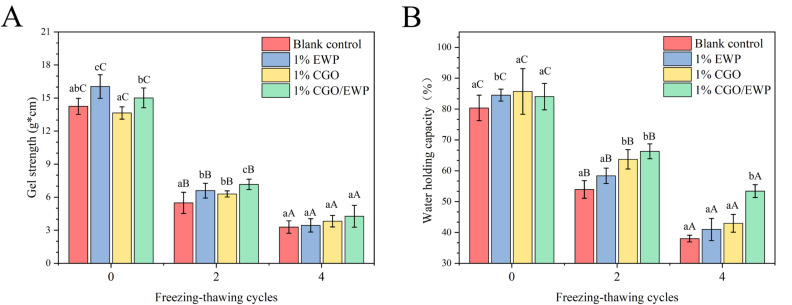
Changes of gel strength (**A**) and water-holding capacity (**B**) in the heat-induced gel of *Culter alburnus* myofibrillar protein added with EWP, CGO or CGO/EWP during freezing–thawing cycles. A significant difference (*p* < 0.05) between different experimental groups in the same freezing–thawing cycle is expressed by different lowercase letters (a–c), and a significant difference (*p* < 0.05) between different freezing–thawing cycles in the same experimental group is expressed by different uppercase letters (A–C).

Gel strength is one of the major indicators for the MP gelation properties and highly related to the three-dimensional gel network [35]. As shown in Figure 4, in the 0 F-T-C, the gel strength was significantly (*p* < 0.05) higher in the EWP group than in the CGO/EWP group. Meanwhile, the two groups were significantly (*p* < 0.05) higher than the CGO and blank groups in gel strength. This was consistent with the G′ and G′′ results in the 0 F-T-C and after repeated F-T-Cs (Figure 3), further supporting that EWP could increase the MP viscoelasticity and enhance its gel strength through the filling effect, and the MP gel viscoelasticity would decrease after repeated F-T-Cs. Besides, after the second F-T-C, the gel strength was significantly (*p* < 0.05) higher in the EWP and CGO groups than in the blank group, suggesting that adding either EWP or CGO alone could delay the degradation of MP gel properties, but the effect is not as satisfactory as the CGO/EWP mixture, probably due to their entirely different mechanisms. Specifically, during repeated F-T-Cs, CGO reduces the gel-forming ability by stabilizing the structure (Figure 2) and suppressing the oxidative deterioration of MP functional groups (Figure 1), while EWP improves the deterioration of gel-forming ability by unfolding into molten globular protein as a filler during the gel formation process. Furthermore, after the second F-T-C, the gel strength was significantly (*p* < 0.05) higher in the CGO/EWP group than in the EWP or CGO group, probably because CGO/EWP has the combined advantages of both CGO and EWP in cryoprotection, that is, CGO could delay changes in functional and structural properties, and EWP could improve the MP gel strength and network compactness through its gel filling effect during the gel-forming process.

As a direct indicator for MP gel quality, water-holding capacity (WHC) can reflect the MP ability to bind water and its three-dimensional network integrity. As shown in Figure 4, after repeated F-T-Cs, the WHC significantly (*p* < 0.05) decreased in all the four groups, which was associated with changes in the intermolecular interaction, producing a negative effect on the formation and stability of the gel network [3,36]. However, in the second F-T-C, compared with the blank group, adding EWP, CGO, or CGO/EWP significantly (*p* < 0.05) curbed the decline of WHC. Moreover, the gel WHC was significantly (*p* < 0.05) higher in the CGO and CGO/EWP groups than in the EWP group, probably because CGO contains more hydrogen bonds than EWP, thus binding more water molecules, suggesting the CGO in the CGO/EWP mixture may play a major role in increasing the protein–water molecular interaction in MP gels.

### 3.7. T_2_ Relaxation Time and Proton Density Map in MP Gel

**Figure 5:**
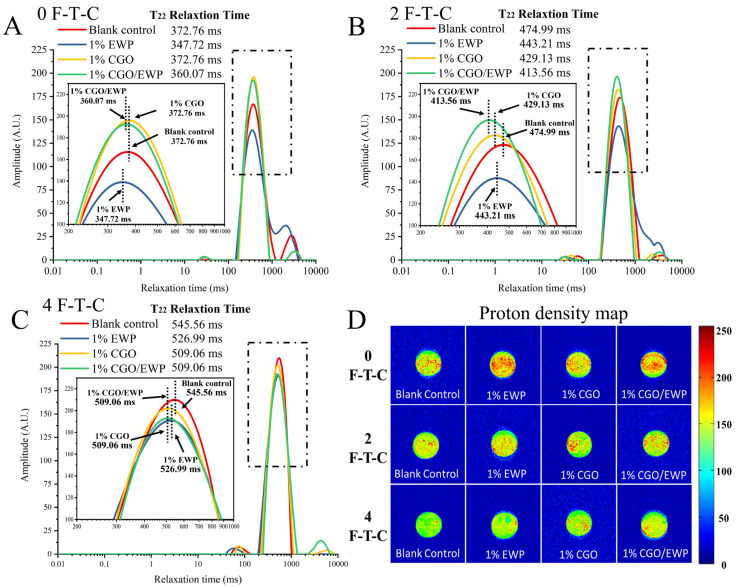
Changes of T_22_ relaxation time (**A**–**C**) and proton image density map (**D**) in the heat-induced gel of *Culter alburnus* myofibrillar protein added with EWP, CGO, or CGO/EWP during freezing–thawing cycles. The blank group was not added with EWP, CGO, or CGO/EWP.

LF-NMR can be used to estimate the water molecular distribution and the protein–water intermolecular interactions in the gel by monitoring the T_2_ relaxation time of hydrogen protons [37]. In Figure 5A–C, the four groups were seen to have three peaks, corresponding to water combined with macromolecules (bound water, T_21_, 0–10 ms), immobile water trapped within the gel network (immobilized water, T22, 100–1000 ms), and water-extra in the gel network (free water, T23, 1000–10,000 ms) [3]. Notably, immobilized water was shown to dominate the water components in the four groups. In the 0 F-T-C, the T_22_ relaxation time was longer in the CGO/EWP (360.07 ms) and EWP (347.72 ms) groups than in the CGO (372.76 ms) and blank (372.76 ms) groups, proving that EWP could enhance the compactness of the gel network through its filling effect and reduce the water mobility. After repeated F-T-Cs, the four groups all showed a varying degree of increase in the T_22_ relaxation time, verifying that the F-T-C could weaken the MP gelation properties and reduce protein–water intermolecular interactions. Compared with the blank group, adding CGO/EWP, CGO, and EWP could curb the increase of T_22_ relaxation time. Furthermore, in the second F-T-C, the relaxation time was shorter in the CGO group (429.13 ms) than in the EWP group (443.21 ms), inferring that, compared with EWP, CGO could more effectively inhibit the exposure of MP hydrophobic groups during repeated F-T-Cs (Figure 2A), thereby delaying the increase of hydrophobic interactions in the MP gel. Additionally, the abundant hydrogen bonds in CGO could also increase the ability of the MP gel to bind water. Moreover, in the 0 F-T-C, the relaxation time was shorter for the EWP group (347.72 ms) relative to the CGO group (372.76 ms), in contrast to a longer relaxation time in the EWP group (443.21 ms) than in the CGO group (429.13 ms) in the second F-T-C. A possible explanation is that EWP is a macro-molecular substance and CGO is a small molecule, which has better stability in F-T-C and could delay the decline of protein–water molecular interaction in MP gels.

In the second F-T-C, the CGO/EWP group (413.56 ms) showed a shorter T_22_ relaxation time than the EWP (443.21 ms) or CGO (429.13 ms) groups, suggesting the better protective effect of CGO/EWP on MP than adding EWP or CGO alone, and the CGO/EWP mixture had the combined advantages of both EWP and CGO. Furthermore, during repeated F-T-Cs, the CGO in the CGO/EWP mixture could delay the exposure of MP hydrophobic groups and restrain water mobility through the formation of hydrogen bonds with water molecules. During the gel-forming process, the EWP in CGO/EWP could be used as a filler in the gel network structure and increase the ability of the MP gel to bind water molecules.

As a technology highlighting water distribution, the proton density map with brighter and redder color indicates more hydrogen protons in the given area [3]. As shown in Figure 5D, after repeated F-T-Cs, the proton image density map of the four experimental groups showed an increase in brightness and a decrease in red area, further showing that F-T-C could weaken the MP gel forming ability, while simultaneously reducing the interaction forces between protein–water molecules and decreasing the water holding ability of gel network. In the 0 F-T-C, the proton imaging results are consistent with the T_22_ relaxation time results. Compared with the blank and CGO groups, the EWP and CGO/EWP groups had more red areas, indicating that adding EWP could increase the compactness of the gel network and improve the ability of the MP gel to bind water. After the second F-T-C, the CGO/EWP group showed more red areas and higher brightness, indicating that adding EWP/CGO could more effectively delay the degradation of MP gel properties.

### 3.8. Selective Solubility in MP Gel

Variations in the selective solubility can reflect changes of intermolecular interaction forces in the heated-induced MP gel. From the microscopic view, the intermolecular interaction between the protein–protein and protein–water molecules drives the gel-forming process of the three-dimensional network, maintaining the gel structure and affecting the gelation properties [38]. Furthermore, ionic and hydrogen bonds were the primary forces for binding water molecules and forming immobilized water [3]. After repeated F-T-Cs, the four groups showed a decrease in the content of ionic bonds and hydrogen bonds, which was consistent with the above conclusions that F-T-C could cause the decrease of WHC (Figure 4) and deteriorate the ability of the MP gel to bind water molecules (LF-NMR, Figure 5). Furthermore, the increased hydrophobic interactions in the MP gel further proved that F-T-C could cause the decrease of the gel viscoelasticity (Figure 3) and strength (Figure 4).

**Figure 6:**
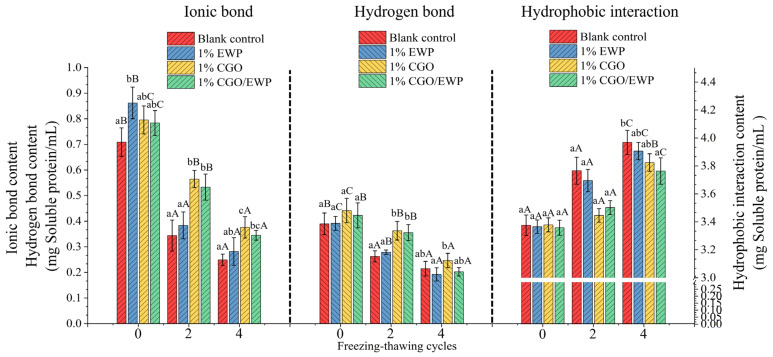
Changes of non-covalent intermolecular interaction in the heat-induced gel of *Culter alburnus* myofibrillar protein added with EWP, CGO, or CGO/EWP during freezing–thawing cycles. The blank group was not added with EWP, CGO, or CGO/EWP. A significant difference (*p* < 0.05) between different experimental groups in the same freezing–thawing cycle is expressed by different lowercase letters (a–c), and a significant difference (*p* < 0.05) between different freezing–thawing cycles in the same experimental group is expressed by different uppercase letters (A–C).

As shown in Figure 6, after the second F-T-C, the contents of hydrogen bonds and ionic bonds were significantly (*p* < 0.05) higher in the CGO and CGO/EWP groups than in the blank group. The ionic bonds are derived from the interaction between the protein/water molecules and the electrostatic repulsion between the protein/protein molecules caused by the negative surface charges [3]. Meanwhile, hydrogen bonds play an important role in protein secondary structure stability and are associated with gel formation rearrangement (α-helix alters β-sheet), thus affecting gelation properties [39]. Therefore, during F-T-Cs, CGO delayed the decrease in the content of ionic bonds and hydrogen bonds in MP gels by enhancing MP structural stability and reducing the exposure of hydrophobic groups inside the MP core. Additionally, CGO contained more hydroxyl groups, which could also interact with water molecules through hydrogen bonds, inhibiting the protein–water intermolecular interaction forces in the MP gel. After the second F-T-C, the content of ionic bonds and hydrogen bonds was also significantly (*p* > 0.05) higher in the CGO and CGO/EWP groups than in the EWP and blank groups, indicating that CGO in the CGO/EWP mixture plays a major role in delaying the decrease of protein–water interaction in MP gels caused by repeated F-T-Cs. Furthermore, the content of ionic bonds was significantly (*p* < 0.05) higher in the EWP group than in the blank group in the 0 F-T-C, but not significantly (*p* > 0.05) different between the two groups after the second F-T-C. Although adding EWP could improve the MP gel strength, it could not produce a sustained effect in inhibiting the decrease of protein–water intermolecular interaction force caused by F-T-Cs. As a result, adding CGO and CGO/EWP would be more effective in curbing the decline of protein–water intermolecular interaction.

The formation of a compact protein gel network structure depends on the ordered exposure of sulfhydryl and hydrophobic groups to the polar environment, allowing the steady expansion of amino acid side chains during the gel-forming process [3,32]. The increase of hydrophobic interaction in the MP gels further confirmed that the weakening of the gel network was mainly caused by the irregular exposure of hydrophobic groups and the increase of surface hydrophobicity (Figure 2) induced by repeated F-T-Cs. After the second F-T-C, the hydrophobic interaction force was significantly (*p* < 0.05) lower in the EWP, CGO, and CGO/EWP groups than in the blank group, indicating that adding EWP, CGO, or CGO/EWP could inhibit the deterioration of the MP gel network caused by repeated F-T-Cs. Additionally, the hydrophobic interaction force was significantly (*p* < 0.05) lower in the CGO and CGO/EWP groups than in the EWP group. Therefore, compared with adding EWP alone, CGO could more effectively delay the increase of hydrophobic interactions in gel by inhibiting the exposure of hydrophobic groups during repeated F-T-Cs.

Collectively, compared with EWP, adding CGO or CGO/EWP can more effectively delay the changes of intermolecular interaction in MP gel, especially between protein–water molecules.

## 4. Conclusions

This study revealed that repeated F-T-Cs can cause the oxidation of sulfhydryl groups, the generation of carbonyl groups, and the exposure of hydrophobic groups inside the protein core, resulting in the deterioration of MP gelation properties, which can be reflected explicitly by the weakening of gel network, viscoelasticity, strength, and WHC properties. Compared with adding EWP or CGO alone, adding CGO/EWP could more effectively delay the deterioration of MP gelation properties during F-T-Cs. Specifically, CGO in the CGO/EWP mixture plays the role of providing hydroxyl groups to interact with the MP peptide chains to enhance its structural stability as well as reduce the exposure of hydrophobic groups and inhibit irregular MP aggregation, thereby delaying the decline of its gel-forming ability caused by protein oxidation and ice crystal growth. During the gel-forming process, EWP in the CGO/EWP mixture plays the major role of filler to improve the weakening of the MP three-dimensional gel network caused by F-T-Cs, thereby increasing the MP gel viscoelasticity and enhancing the gel strength. The data of T_22_ relaxation time and intermolecular interaction force indicated that CGO in the CGO/EWP mixture could not only inhibit the exposure of hydrophobic groups caused by F-T-Cs, but also reduce the water–protein and protein–protein interactions. Moreover, CGO could increase the intermolecular interactions at functional sites due to availability of hydroxyl groups, thereby improving the water binding ability and protecting the MP gel properties. Overall, this study has explored the effects and related mechanisms of combining oligosaccharides with egg white protein as a low-calorie cryoprotectant in preventing the oxidative, nutritional, and sensory deterioration of fish muscle protein products under repeated freezing–thawing cycles or long-term frozen storage, providing useful information for the potential use of CGO/EWP for industrial preservation of seafood and fish products.

## Data Availability

The data presented in this study are available in article.
